# Evaluation of Paracervical Block and IV Sedation for Pain Management during Hysteroscopic Polypectomy: A Randomized Clinical Trial

**DOI:** 10.1155/2017/5309408

**Published:** 2017-06-06

**Authors:** Zahra Asgari, Maryam Razavi, Reihaneh Hosseini, Masoumeh Nataj, Mahroo Rezaeinejad, Mahdi Sepidarkish

**Affiliations:** ^1^Department of Obstetrics and Gynecology, Arash Women's Hospital, Tehran University of Medical Sciences, Tehran, Iran; ^2^Department of Epidemiology and Reproductive Health, Reproductive Epidemiology Research Center, Royan Institute for Reproductive Biomedicine, ACECR, Tehran, Iran

## Abstract

**Background:**

The aim of this study was to compare the effectiveness of paracervical block (PB) and IV sedation (IVS) on women's pain perception during operative hysteroscopy.

**Methods:**

A total of 84 patients with uterine polyps were randomized to either PB or IV sedation or general anesthesia (GA) as control group. In PB group, the patients received oral diazepam 10 mg and 100 mg diclofenac Na suppository 60 min before surgery and 10 cc of 2% buffered lidocaine was injected at cervix. Conscious sedation was performed with the IV administration of 2-3 mg/kg/h propofol 1% and midazolam 0.02 mg/kg and fentanyl (1-2 *μ*g/kg) with o2 4-5 lit/min via face mask.

**Results:**

There were no significant differences between groups on VAS score at 3 hours after operation (PB: 1.22 ± (1.31), IVS: 1.10 ± (1.68), GA: 1.29 ± (2.03), *P* = 0.671) and during recovery (PB: 0.85 ± (1.06), IVS: 0.68 ± (1.33), GA: 1.21 ± (2.04), *P* = 0.458). There was no difference between PB (3.33 ± (2.81)) and IVS (2.31 ± (2.63)) groups at hysteroscopy (*P* = 0.182). Patients undergoing IVS reported lower VAS score than PB group at dilation and curettage, although the difference was not statistically significant (PB: 2.59 ± (1.78), IVS: 1.72 ± (2.34), *P* = 0.051). Moreover, patients undergoing IVS obtained lower VAS score than PB group at polypectomy, while the difference was not statistically significant (PB: 1.81 ± (1.52), IVS: 1.10 ± (1.32), *P* = 0.073).

**Conclusion:**

The finding of the present study revealed that IVS and PB showed the same effect in reducing pain during and after gynecological surgical procedures. The study was registered in Iranian Registry of Clinical Trial with the number IRCT2016031426855N3, on April 28, 2016.

## 1. Background

Overgrowth of endometrium cells in the lining of uterus or cervix is known as endometrial polyp [1]. Hysteroscopic polypectomy, an outpatient surgical procedure performed for the removal of uterine polyps, is a minimally invasive treatment aimed to remove these polyps while keeping the uterus intact. This procedure is performed within the uterine cavity using operative hysteroscopy [2, 3].

With the advance technologies, nowadays, there are finer and miniaturized hysteroscopes available which can be used to perform a wide range of simple surgical operations [4]. These devices allow us to do surgeries easier using sedation or local anesthesia. Several methods have been used for pain reduction [5, 6]. Moreover, multiple studies suggested that the outpatient hysteroscopy is safer and more satisfactory when performing under moderate sedation [7–9].

PB and IVS are commonly used methods in pain reduction during cervical dilatation and uterine interventions (such as hysteroscopic polypectomy, endometrial biopsies, fractional curettage, and suction terminations). In PB, local anesthetic is injected around the cervix to numb nearby nerves [10]. PB has been used by many gynecologists for uterine intervention; however, its effectiveness and safety are still controversial [10]. In IVS, the anesthetic drug is injected to the blood vessel in doses lower than general anesthesia (GA). Typically the sedation level that is accomplished with IVS is considered as conscious or deep sedation, as conscious sedation is a minimally depressed level of consciousness providing an independent and constant breathing for patients and it makes them able to respond to physical stimulation and verbal command. Several studies have suggested that we do not need any anesthesia during diagnostic hysteroscopic procedures [11–17]. In a study conducted by Centini et al., it has been concluded that moderate sedation together with a PB will reduce pain perception and the operative time will decrease [18]. Cooper et al. reported outpatient polypectomy which uses local anesthesia is more safe and time and cost effective, although the inpatient procedure under GA was more successful noting that patients undergoing outpatient surgery were twice more likely to undergo another surgery after two years [19].

Despite the fact that many studies have been conducted regarding the safest and more time and cost effective method for pain reduction during uterine interventions, there is still controversy over the best procedure. The goal of this randomized clinical trial is to evaluate the efficacy and effects of the PB and IVS applied for pain reduction among patients undergoing hysteroscopic polypectomy.

## 2. Methods

### 2.1. Patient Inclusion and Randomization

This study was conducted at Arash Hospital, Tehran, Iran, between April 2016 and June 2016. Arash Hospital is a General Women's Hospital located in Tehranpars, an eastern suburb of Tehran in Iran. The study was designed as a single-center, randomized, parallel-group, controlled trial in accordance with Consolidated Standards of Reporting Trials (CONSORT) guidelines [20].

The participants were women aged over 18 years with abnormal uterine bleeding and a report of the polyp in transvaginal sonography who were undergoing operative hysteroscopy for endometrial polyps were selected after anesthesiologic evaluation in a prehospitalization regimen.

The study was performed in accordance with the Declaration of Helsinki and was approved by the ethical committee of our hospital [21]. Written, informed consent was obtained from the patients before any study-related tests were done.

Ethics approval was obtained from the Tehran University of Medical Sciences Clinical Research Ethics Board. The study was registered in Iranian Registry of Clinical Trial (http://www.irct.ir) by the number of IRCT2016031426855N3.

We enrolled female patients who had endometrial polyps, American Society of Anesthesiologists (ASA) physical statuses I-II and age > 18 years. Exclusion criteria included severe cardiovascular diseases, allergy to local anesthetics, pathologies connected with abdominopelvic pain that could confuse the perception of pain directly related to the procedure (e.g., endometriosis), being unable to comprehend visual analog scale, and patients in disagreement with the study protocol.

After their consent was obtained, patients were randomly assigned in three groups, using a random number sequence, generated with a computer-generated randomization scheme, according to a randomized block design. The block size was 6. Allocation concealment was maintained by having procedure indicator cards inside a set of numbered opaque sealed envelopes. Trial's epidemiologist, who was not, involved in the selection and allocation of patients prepared, coded, and sealed opaque envelopes. Patients were allocated treatment by the author opening the next numbered envelope, after screening, in the presence of the patient.

### 2.2. Study Protocol

Following our standard practice a 20 G cannula needle was inserted in each patient. In the GA group, general anesthesia was induced with midazolam (0.02 mg/kg) (Chimidarou Industrial Company, Tehran, Iran), propofol (1% 1–2.5 mg/kg) Lipuro 1% (B. Braun Melsungen AG, Germany), and fentanyl (1-2 *μ*g/kg) (Aburaihan Co., Iran) and a laryngeal mask (Nanjing Hong An Medical Appliance, silicone numbers 3-4, Nanjing, China) was applied. Each patient, in the gynecologic position, was connected to a Drager Infinity Delta ventilator (Drager Fabius GS, Germany) in a volume-controlled ventilation mode. The anesthesia was maintained with a continuous intravenous infusion of propofol (1%, 100–200 *μ*g/kg/min) and oxygen in air. Fentanyl was repeated during anesthesia according to the attending anesthetist. At the end of the operative procedure, anesthetic infusion was stopped, and the laryngeal mask was removed when the patient started to breathe spontaneously.

*In Group B (PB)*. The patients were received oral diazepam 10 mg as-anxiolytic and 100 mg diclofenac Na suppository as preemptive analgesia, about 60 min before surgery in the ward. In the operation room, 10 cc of 2% buffered lidocaine was injected at 4 and 8 o'clock position at the junction of cervix and vagina at an estimated depth of 1 cm with using a 22-gauge spinal needle. Intermittent aspiration was performed before and during injection to ensure that paracervical blood vessels were not punctured. 

*In Group C (IVS)*. Conscious sedation was performed 10 minutes before surgery with the IV administration of 2-3 mg/kg/h propofol 1% and midazolam 0.02 mg/kg and fentanyl (1-2 *μ*g/kg) with o2 4-5 lit/min via face mask.

The procedure performed by a gynecological surgeon in follicular phase of endometrium, according to the standard practice of our institute, with the patient in the gynecological position. The technique used for diagnostic hysteroscopy was standardized and all procedures were carried out by the same surgeon. Hysteroscopy was performed by first placing a tenaculum on the anterior lip of the cervix at 12 o'clock to stabilize the cervix. A 0.9% normal saline distention medium was used, while the hysteroscope was slowly slid in to the uterine cavity and the pressure for distending the cavity was supplied by a pump up to 0.1 bar. Instruments used were a Hamou 2.9-mm hysteroscope with a 30-degree fore-oblique lens and a 4 mm diagnostic sheath (Karl-Storz GmbH & Co. KG, Tuttlingen, Germany). The appearance of the uterine cavity was recorded for diagnostic purposes. The speculum was removed once the scope had been inserted through the cervical canal.

Small endometrial polyps were removed using grasper forceps introduced down the operating channel of the Versascope™. Larger polyps were first divided into smaller pieces using the scissors and the fragments were then removed using grasper forceps. All tissues were sent for histological diagnosis.

### 2.3. Primary Endpoint and Power Analysis

In IVS and PB groups, the patients were asked to mark their pain on a 10 cm visual analog scale (VAS) at six points during the procedure: hysteroscopy, dilation and curettage, PB administration (if in this arm of the study), polypectomy, three hours after procedure, and recovery. Visual analog scale (VAS) is a robust and reliable instrument which has been regularly used in similar trials by multiple investigators for different procedures [22]. In GA group, they only were asked in recovery and after procedure. The study was designed to have 80% power to detect a 35% difference in pain scores on visual analog scale, with two-sided alpha levels of 0.05. Using sample size calculation for independent proportions, the minimum number of participants in each group should be 28 (total 84 participants).

### 2.4. Statistical Analyses

All data analyses were conducted using SPSS version 16.0 for Windows (SPSS Inc., Chicago, IL, USA). Descriptive statistics for continuous variables were presented as mean ± standard deviation (SD) and for categorical variables as numbers (percentage). Nonparametric tests were chosen because study variables were not normally distributed (Shapiro-Wilk test; all *P* < 0.05). The baseline characteristics of the three groups were compared using Kruskal-Wallis* H* test (followed by post hoc Dunn's test) for continuous variables and the Chi-square test for categorical variables. Moreover, the VAS score for pain and total time operation between groups was compared using Kruskal-Wallis test and Mann–Whitney. All statistical tests were two-sided and the level of statistical significance was set at 0.05. All analyses were performed on an intent-to-treat basis. The conduct and analysis of the trial adhered to the 2010 CONSORT guidelines.

## 3. Results

### 3.1. Participant Characteristics

**Figure 1:**
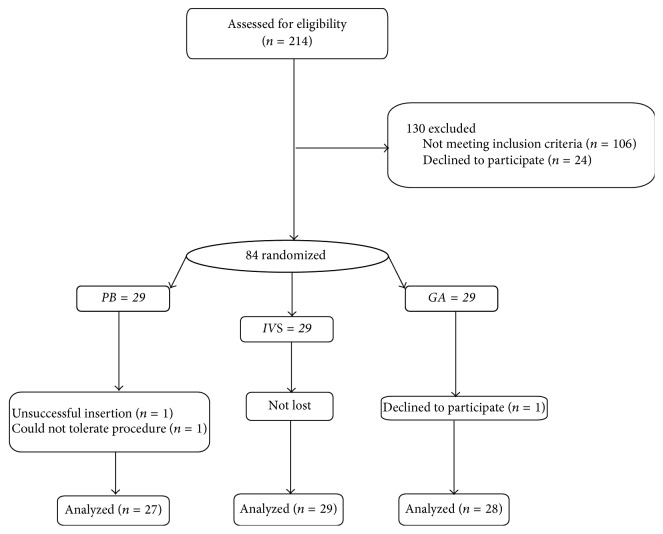
Flowchart showing participants recruitment.

Following recruitment, 214 evaluations yielded the desired sample size of 84 randomized participants (Figure 1). At baseline, the demographic and clinical characteristics were comparable across three groups (all *P* > 0.05) (Table 1). The mean age of the women was 40.28 ± (7.52) years, 28.6% of women had at least 2 polyps, and the mean size of polyps was 2.39 ± (3.53) cm.

### 3.2. Primary Outcomes

**Figure 2:**
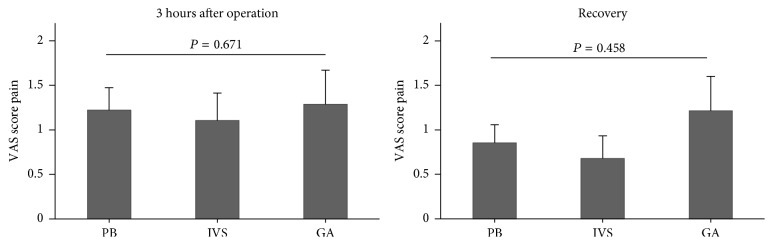
The results of reported pain experienced at 3 hours after operation and recovery. PB: paracervical block; IVS: IV sedation; GA: general anesthesia. The Kruskal-Wallis test was used to compare between groups.

The Kruskal-Wallis test was used to examine the difference of VAS score at 3 hours after operation and recovery between three groups. As shown in Figure 2, there were no significant differences between groups on VAS score at 3 hours after operation (PB: 1.22 ± (1.31), IVS: 1.10 ± (1.68), GA: 1.29 ± (2.03), *P* = 0.671) and during recovery (PB: 0.85 ± (1.06), IVS: 0.68 ± (1.33), GA: 1.21 ± (2.04), *P* = 0.458).

**Figure 3:**
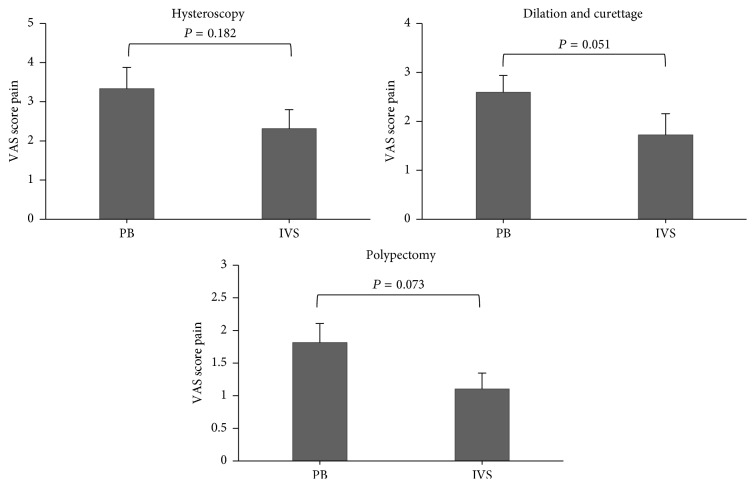
The results of reported pain experienced at hysteroscopy, dilation and curettage (DC), and polypectomy in study groups. PB: paracervical block; IVS: IV sedation. The Mann–Whitney test was used to compare between groups.

Furthermore, the Mann–Whitney test was used to examine the difference of VAS score between PB and IVS at hysteroscopy, dilation and curettage (DC), and polypectomy. According to the results, there was no difference between PB (3.33 ± (2.81)) and IVS (2.31 ± (2.63)) groups at hysteroscopy (*P* = 0.182). Patients undergoing IVS reported lower VAS score than PB group at dilation and curettage, although the difference was not statistically significant (PB: 2.59 ± (1.78), IVS: 1.72 ± (2.34); *P* = 0.051). Moreover, patients undergoing IVS obtained lower VAS score than PB group at polypectomy, while the difference was not statistically significant (PB: 1.81 ± (1.52), IVS: 1.10 ± (1.32); *P* = 0.073) (Figure 3).

### 3.3. Secondary Outcomes

There was no statistical difference in the operating time between three groups (PB: 28.48 ± (10.03), IVS: 24.86 ± (12.29), GA: 27.57 ± (7.92), *P* = 0.245). In all the three groups, no side effects were observed among the patients.

## 4. Discussion

The finding of the present study revealed that, compared with IVS, PB showed the same effect in reducing pain during surgical procedures, including hysteroscopy, dilation and curettage, and polypectomy. The mean pain intensity was the same after 3 hours of the procedure and at the time of recovery between the three techniques of general anesthesia, IVS, and PB.

In a similar study, Centini in Italy compared GA with combination of IVS and PB. In the study, the tool of BPI (Brief Pain Inventory) was used because the pain score based on VAS scale was reported less than 3 in all patients. Finally, they concluded that women who had a combination of two methods of PB and IVS felt significantly less pain on daily activities [18]. In addition to differences in the types of interventions, it seems that the differences between the findings of the study and the present study were use of various pain assessment tools. They used IV fentanyl and propofol for sedation in PB group. It is predictable that this strong analgesia will alleviate patient pain, but there are some challenges. First of all, we try to improve PB methods to make it suitable for office hysteroscopy, while it is impossible to use parenteral analgesia at office. Furthermore, we are going to find a less expensive, more effective method with shorter recovery time. Hence, this study was designed to separate IV sedation group from PB ones. Since PB is a little painful for patients, we provided a light nonparenteral sedation with an oral Benzodiazepine and a rectal nonsteroid anti-inflammatory drug (NSAID). Two studies similar to the present study used the VAS scale and reported similar findings. In multicenter clinical trial that was conducted in 2003 by Guida et al., 166 women were under operative hysteroscopy. They compared local PB anesthesia and conscious sedation using atropine 0.5 mg, fentanyl 25 *μ*g, and midazolam 2 mg. No significant difference was observed between the two groups in any of the times [3]. Although they used the same drugs as our study except for propofol, they reported lower pain scores. It may reveal that propofol has no benefit for pain alleviation and should be omitted from conscious sedation protocols. It can be a matter for future studies. In another study that was conducted in 2011 by Thongrong et al., they evaluated PB and intravenous morphine in 64 women (two groups of 32 people) who were candidate for curettage. The mean of pain was not significantly different between the two groups [23].

In a systematic review conducted by Cooper et al. in 2010, the effect of local anesthesia (paracervical and intracervical) in order to control pain during outpatient hysteroscopy was investigated. Of 20 trials (2581 participants), 15 high-quality studies were entered to the final meta-analysis. The results showed that intracervical anesthesia (SMD: −0.36; 95% CI: −0.61 to −0.1) and PB (SMD: −1.28; 95% CI: −2.22 to −0.35) affect pain during outpatient hysteroscopy. The intervention in most studies was diagnostic and one of the reasons for the difference between that review and the present study is the use of operative hysteroscopy [24].

In a systematic review in 2013, databases, such as Cochrane Central Register of Controlled Trials (CENTRAL) (The Cochrane Library 2013, Issue 8), MEDLINE (1966 to August 2013), EMBASE (1980 to August 2013), and reference lists of articles, were searched. Finally, 26 articles were included in the meta-analysis. Interventions listed in the review included a wide range of procedures such as endometrial biopsy, fractional curettage, and vacuum aspiration, and the researchers concluded that according to the available evidence the superior or inferior efficiency and safety of PB or other alternative methods of anesthesia cannot be decided. In 10 of the 26 studies in the systematic review, PB was compared with placebo. Uterine interventions included hysteroscopy, endometrial biopsy, fractional curettage, and vacuum aspiration. In these studies, for local anesthesia, lidocaine, chloroprocaine, and xylocaine were used. The pooled results from 10 studies revealed that PB had no effect on pain of speculum insertion (Standard Mean Difference: −0.2; 95% CI: −0.35 to 0.74). PB also did not reduce pain of tenaculum placement (SMD: 0.7; 95% CI: −2.26 to 0.86). The pain of dilating cervix was reduced by PB (SMD: −0.39; 95% CI: −0.72 to −0.07). Pain during uterine interventions was reduced (SMD: −0.74; 95% CI: −1.19 to −0.28). The results of analysis of 10 studies imply very diverse heterogeneity of the studies (*I*^2^: 0.86, *P* < 0.001). Subgroup analysis showed that PB in reducing pain in endometrial biopsy and suction aspiration is more effective than other procedures. The results showed that PB has no effect in reducing postoperative pain and shoulder pain. In the study, six of 26 studies compared PB with no anesthesia. In any of six studies, PB had no effect on pain during and after the procedure. Of the 26 studies, five studies compare PB impact with other methods of regional anesthesia. All of five studies reported PB effect of the pain during and after the procedure nonsignificantly. Finally, 6 studies also compared PB with other pain systemic analgesia and the results of the meta-analysis revealed very diverse heterogeneity and there was no evidence of the effectiveness of PB on pain during and after procedure. In the study, there was no study retrieved on comparison of PB with general anesthesia [25].

If we consider GA as an optimal method for reducing pain during procedure, other procedures should be compared with it. In IVS method, we use lower dose of drugs and it does not need laryngeal mask. These two points reduce the costs and side effects besides recovery time. Therefore, according to our findings which imply acceptable pain relief using IVS, it can substitute GA for operative hysteroscopy in many cases.

Also these findings suggest that PB is a good and effective method which provides the possibility of outpatient or office operative hysteroscopy in many patients and offers an alternative for anesthesia in patients with serious medical condition.

When efficacy of PB was investigated, other variables influencing pain were taken into account, but the variables have been neglected in previous studies. These variables can include the skill of the surgeon and patient characteristics and be used in diagnostic or therapeutic procedure. However, in the previous trials, random allocation equally distributes confounding variables between groups; comparison of the effects of different studies together the variables can justify the differences.

In conscious IVS and PB, we have other benefits such as verbal communication between the patient and the surgeon during surgery, more emotional support, and consequently reduction in anxiety [23]. In the present study, however, no statistically significant difference was observed between the two methods of PB and IVS in different procedures, but the lack of statistical significance was very fragile cross-border.

## 5. Conclusion

Finding of the present study revealed that IVS and PB are being able to reduce pain during and after hysteroscopic procedures and can be reasonable substitutes for GA. More studies with more sample sizes may highlight this difference.

## Figures and Tables

**Table 1:** Characteristics of the patients in study groups.

	PB (*n* = 27)	IVS (*n* = 29)	GA (*n* = 28)	*P* value
Age (years), mean ± SD	38.48 ± 5.71	41.44 ± 8.15	40.92 ± 8.38	0.308
Gravid (%)				
0	5 (18.5)	6 (20.7)	5 (17.9)	0.273
1	10 (37.0)	4 (13.8)	5 (17.9)	
>2	12 (44.4)	19 (65.5)	18 (64.3)	
Number of CS, *n* (%)				
0	14 (51.9)	20 (69.0)	18 (64.3)	0.673
1	9 (33.3)	5 (17.2)	7 (25.0)	
>2	4 (14.8)	4 (13.8)	3 (10.7)	
Number of NVD, *n* (%)				
0	18 (66.7)	13 (44.8)	15 (53.6)	0.543
1	2 (7.4)	5 (17.2)	3 (10.7)	
>2	7 (25.9)	11 (37.9)	10 (35.7)	
Number of polyps, *n* (%)				
1	19 (70.4)	21 (72.4)	20 (71.4)	0.986
>2	8 (29.6)	8 (27.6)	8 (28.6)	
Size of polyps (mm), mean ± SD	2.20 ± 3.39	2.95 ± 4.65	2.02 ± 2.10	0.579
Menopause, *n* (%)				
No	26 (96.3)	26 (89.7)	25 (89.3)	0.572
Yes	1 (3.7)	3 (10.3)	3 (10.7)	

PB: paracervical block; IVS: IV sedation; GA: general anesthesia, CS: Cesarean section; NVD: natural vaginal delivery.

## Abbreviations

PB: Paracervical block
IVS: IV sedation
GA: General anesthesia
VAS: Visual analog scale.
